# The Assassination of the President

**Published:** 1901-10

**Authors:** 


### *EDITORIAL NOTES AND COMMENTS.*

The Business office of THE JOURNAL-RECORD is No. 64 Marietta Street.

The Editorial office is 318-319-320 The Prudential.

Address all Business communications to Dr. M. B. Hutchins, Mgr.

Make remittances payable to THE ATLANTA JOURNAL-RECORD OF MEDICINE.

On matters pertaining to the Editorial and Original Communications address Dr. Bernard Wolff, Atlanta.

Reprints of original articles will be furnished at cost price. Requests for the same should always be made *on the manuscript.*

We will present, post-paid, on request, to each contributor of an original article, twenty (20) marked copies of THE JOURNAL-RECORD containing such article.

### THE ASSASSINATION OF THE PRESIDENT.

IT is always sad to see a useful life untimely ended, and the sadness that pervades the country from the foul murder of President McKinley is almost lost in the feverish desire for retaliation upon the wretch who perpetrated the deed and all of his kind. Though differing from the vast bulk of Southern men in politics, there are few among them who did not admire McKinley for his serene, majestic character, and who do not feel the shock of his death with almost the intensity of a personal loss. That such a purpose full, patriotic and pure life should have been uselessly sacrificed to the silly vengeance of an offshoot of a rotten European civilization is hard to bear, and doubly so when there are so many incumbents of political office whose taking off could be borne with so much Christian fortitude and resignation.

> "Patroclus liegt begraben
> Thersites kommt zürück."

Medically speaking, though unusual, there was nothing mysterious about the cause of the President's death. The course of the bullet was gangrenous from destruction of tissue of low reparative power, and the negative result, as regards poison of the chemical analysis of the coating of the bullet, was nothing more than what every one expected. There is no poison, organic or inorganic, which is capable of producing the effects noted in the clinical course of the President's illness and displayed by the autopsy.

Though the treatment of the case was orthodox and in accord with the canons of modern surgery, there were many, with the direful experiences of the surgeons in the Spanish-American and Anglo-Boer wars with laparotomy for gunshot wounds of the abdomen fresh in mind, who discounted the optimistic expressions of the press in regard to the prognosis, and confidently expected a fatal issue.

The assassin takes no chances, and his victim, being utterly unsuspecting and defenceless, is at his mercy so that he may make the stroke sure and certain.

Lincoln was shot in the head and died within an hour after the wound was given. Garfield was shot in the back, the ball traversing the body of one of the dorsal vertebrae and his death was due to sepsis and pneumonia several months later. Henry IV was stabbed to the heart, as was also the Empress of Austria. Alexander II was blown to tatters. In every case the treacherous knife or bullet showed a malevolence that accorded well with that of the hand which sent it, and did its work without bungling.

The country is tired of having its bravest and its best sacrificed to a wretched foreigner's murderous whim, and the period is ripe for treating the reds of anarchy to a taste of the fagot and the stake for the glory of the Lord and the preservation of American citizens.

Hanging is too cheap and banal a punishment for the wretched Polack. A more refined retaliatory expiation for his crime would consist in solitary confinement in a darkness which would be illumined by a single shaft of light directed upon a death mark of the President fixed to the wall out of reach of the hand of the mowing idiot that would be found there before a month was out. The constant reminder of his victim would offer food for reflection, and this form of reflection is not good for the mind.

